# The intersection of health and wealth: association between personal bankruptcy and myocardial infarction rates in Canada

**DOI:** 10.1186/s12889-016-2705-x

**Published:** 2016-01-13

**Authors:** Anamaria Savu, Donald Schopflocher, Barry Scholnick, Padma Kaul

**Affiliations:** 1 Canadian Vigour Center, University of Alberta, Edmonton, AB T6G 2E1 Canada; 2 Faculty of Nursing, University of Alberta, Edmonton, AB T6G 1C9 Canada; 3 Alberta School of Business, University of Alberta, Edmonton, AB T6G 2R6 Canada

**Keywords:** Canada, Acute myocardial infarction, Personal bankruptcy, Socio-economic characteristics, Ecological study, Correlation, Cross-lagged structural equation model

## Abstract

**Background:**

We examined the association between personal bankruptcy filing and acute myocardial infarction (AMI) rates in Canada.

**Methods:**

Between 2002 and 2009, aggregate and yearly bankruptcy and AMI rates were estimated for 1,155 forward sortation areas of Canada. Scatter plot and correlations were used to assess the association of the aggregate rates. Cross-lagged structural equation models were used to explore the longitudinal relationship between bankruptcy and AMI after adjustment for socio-economic factors.

**Results:**

A cross-lagged structural equation model estimated that on average, an increase of 100 in bankruptcy filing count is associated with an increase of 1.5 (*p* = 0.02) in AMI count in the following year, and an increase of 100 in AMI count is associated with an increase of 7 (*p* < 0.01) in bankruptcy filing count.

**Conclusions:**

We found that regions with higher rates of AMI corresponded to those with higher levels of economic and financial stress, as indicated by personal bankruptcy rate, and vice-versa.

## Background

Over-indebtedness and health likely have a reciprocal impact, and the connection between them is complex. The debt-health interplay has been described as a vicious cycle leading people into “a poverty trap, i.e., deteriorated health (due to indebtedness) induces job loss which results in even higher relative debt burdens” [1]. Understanding the relationship between debt and health, specifically heart health may therefore be important for identifying policies that are required to break a negative social cycle.

Acute myocardial infarction (AMI), the event commonly known as heart attack, happens when blood stops flowing properly to part of the heart and the heart muscle is injured due to insufficient oxygen. Risk factors for AMI include age, smoking, high blood pressure, diabetes or obesity, and mental stress [2].

AMI is expected to be a health condition involved in the debt-health interplay for several reasons. First, over-indebtedness is a mental stress inducer, and, as mentioned, mental stress is an established risk factor for AMI. Second, AMI could be followed by complications like heart failure, atrial fibrillation or a subsequent heart attack, which in turn could lead to unemployment, loss of income and financial strain.

The current literature on the relationship between debt and heart health is sparse. Accordingly, using data from all Canadian provinces, except Quebec (population of 17.9 million people aged 20 years and over in 2006), we conducted an ecological study to assess not only the cross-sectional relationship but also the longitudinal relationship between debt and heart health.

## Methods

### Data

We used three databases: (1) 2006 Census of Canadian population [3], (2) the Canadian Institute of Health Information Discharge Abstract Database (DAD) on hospitalizations to acute care facilities in all Canadian provinces except Quebec [4], and (3) data on annual counts of insolvencies filed by Canadian individuals [5]. Additional details on the linked databases are presented in Table 1.

**Table 1:** Databases linked for the study

	2006 Census of Canadian population	Discharge abstract database metadata	Annual counts of insolvency filings by Canadian residents
Source	Statistics Canada	Canadian Institute for Health Information	Office of the Superintendent of Bankruptcy Canada
Type	Cross-sectional	Longitudinal	Longitudinal
Time of collection	May 16, 2006	Discharge date between April 1, 2001 and March 31, 2010	January 1, 1994 to December 31, 2009
Information collected	Socio-economic characteristics of Canadian population aggregated at FSA level	Patient scrambled unique identifier, FSA, age and gender, admission and discharge dates, diagnoses and procedures for hospitalizations at acute care facilities in Canada	Counts of insolvency filings per postal code and calendar year separated into bankruptcy and proposal for debt restructuring
Variables/records selected	Population aged 20 years and over, median household income, home ownership, elderly 65 years and over, being the head of a single-parent family, being without a high-school education, being with a university degree, employment rate and unemployment rate	Hospitalizations for AMI as main diagnosis (International Classification of Diseases-9th Revision: 410.xx or 10th Revision: I21.xx)	Counts of BKC filings
FSA in use* at some point between Jan 1st, 2002 and Dec 31st, 2009	1,207 comprising 18,007,300 population aged 20 years and over in 2006	316,393 AMI events	455,337 BKC filings
FSA in use* at any time between Jan 1st, 2002 and Dec 31st, 2009	1,155 comprising 17,935,425 population aged 20 years and over in 2006	315,011 AMI events	453,177 BKC filings

*as assigned by Canada Post for Canadian addresses outside Quebec and Territories [6]; FSA (forward sortation area); AMI (acute myocardial infarction); BKC (bankruptcy)

### Availability of data information

2006 Census of Canadian population is collected and maintained by Statistics Canada. To prevent the confidentiality of the 2006 Census respondents, population-count data are released for areas with at least 40 persons and socio-economic (income) data are released for area with at least 250 persons. Census data are made available publicly and to libraries of Canadian universities in various aggregate databases. The 2006 Census data at forward sortation area (FSA) level used in our study were downloaded from the Data Library of University of Alberta.

DAD Metadata were requested and received from the Canadian Institute of Health Information. DAD records demographic, administrative and clinical data on all hospitalizations at acute care inpatient facilities. To prevent patient identification, each patient is assigned a unique scrambled patient identifier and has his/her address identifier restricted to the first three digits of his/her residential postal code. Ethics approval for secondary use of de-identified acute myocardial infarct data was received in 2008 and has been kept current from the Health Research Ethics Board – Health Panel (REB 3) of the University of Alberta.

Data on insolvency filings by Canadian individuals were provided by the Office of the Superintendent of Bankruptcy, Government of Canada. All data related to individual bankruptcy filings are publicly available because every bankruptcy filing is a legal proceeding before the courts. Thus no ethics approval, (e.g., related to issues of confidentiality), is required for the use of this data.

Analysis data can be made available by the authors, upon request.

### Data aggregation

Our unit of analysis was the FSA. The FSA is a geographical region in which all postal codes (six-character strings) start with the same three characters. The average number of households served by an FSA is 8,000 but the number can range from 0 to 60,000. Our study included all FSAs with non-missing and non-zero population counts in all Canadian provinces, except Quebec, in effect throughout the entire study period Jan 1st, 2002 – Dec 31st, 2009 [6].

There were 1,155 FSAs included in our analysis. The FSAs selected in our study had in 2006 between 250 and 80,025 people aged 20 years or over. For each FSA *i* we extracted the count of population 20 years and over, *P*_*i*_, in 2006, and several socio-economic characteristics (median household income, employment rate, education attainment, home ownership and being the head of a single parent family) from 2006 Census of Canadian population [3]. The selected socio-economic characteristics are factors incorporated in previous socio-deprivation indices [7, 8]. Hospital admissions of patients aged 20 and over residing within the selected 1155 FSAs were clustered into hospitalization episodes (i.e., uninterrupted time intervals of hospitalization days). Hospitalizations with a most responsible diagnosis of AMI with a discharge date between Jan 1st, 2002 and Dec 31st, 2009 were selected as indicators of AMI events. The calendar year of the episode discharge date was used as the calendar year of the AMI episode. Multiple AMI episodes of the same patient in the same year were counted only once. For each year [*t*] and each FSA *i* we identified the number of AMI events *NM*_[*t*]*i*_. From Insolvency filing data we extracted for each year [*t*] and each FSA *i* the number of personal bankruptcy (BKC) filings *NB*_[*t*]*i*_.

AMI and BKC counts were changed into rate estimates as follows: (1) ${M}_i=\frac{{\displaystyle {\sum}_{\left[t\right]}}N{M}_{\left[t\right]i}}{8{P}_i}$ and ${B}_i=\frac{{\displaystyle {\sum}_t}N{B}_{\left[t\right]i}}{8{P}_i}$ as rates over the 8-year time interval from 2002 to 2009 and (2) ${M}_{\left[t\right]i}=\frac{N{M}_{\left[t\right]i}}{P_i}$ and ${B}_{\left[t\right]i}=\frac{N{B}_{\left[t\right]i}}{P_i}$ as rates in year [*t*] for FSA *i*. Because FSA population counts were available for 2006 only, we used these as population counts in all years of the study period. We organized the data in two ways: (1) aggregate data that enclosed AMI rates and BKC rates over the 8-year time interval and (2) panel data that enclosed yearly AMI rates and BKC rates. Both 8-year and yearly rates were reported per 1000 person-years.

### Statistical analysis

Descriptive statistics (mean, standard deviation) were calculated for the 8-year, as well as, yearly AMI and BKC rates and socio-economic factors. Pearson correlation coefficients were calculated to identify associations between AMI and BKC rates, as well as between the socio-economic factors and AMI or BKC rate, and to identify potential socio-economic confounders of the association between AMI and BKC.

Socio-economic variables were standardized to mean zero and variance one, across the 1,155 FSAs. Principal component analysis [9] was used to construct 3 new uncorrelated variables from the 8 standardized census variables. A VARIMAX rotation was applied on the uncorrelated variables to better align them with the census variable and improve their interpretability. We refer to the 3 principal components as education, material wealth and working-age population, and denote their values for FSA *i* by *E*_*i*_, *W*_*i*_, *A*_*i*_.

We estimated the longitudinal association between AMI and BKC by fitting several longitudinal SEM on the panel data that incorporated both temporal relationships AMI × Following-year BKC and BKC × Following-year AMI.

**Fig. 1:**
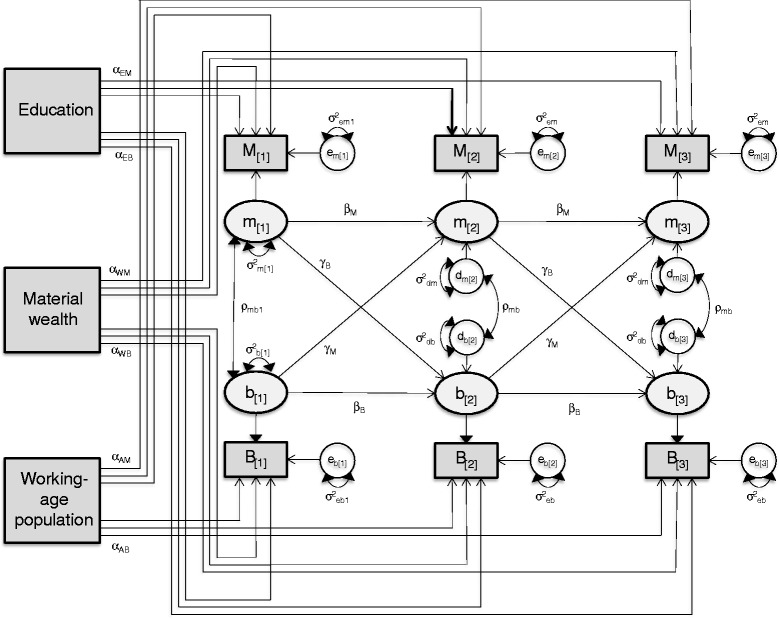
Diagram of SEM for AMI and BKC rates. Path diagram of cross-lagged structural equation model for yearly AMI and BKC rates

The reference model was a cross-lagged model with measurement errors and time-independent covariates [10–14]. The first three waves of the reference model are shown in Fig. 1. The model used the observed yearly AMI and BKC rates as proxies for unobserved heart health and over-indebtedness levels, and further assumed that heart health and over-indebtedness levels incorporated effects of socio-economic variables and latent (unobserved) variables independent of socio-economic factors. Specifically, the system of AMI and BKC rates, measured across years, is explained by the model as follows: for each year [*t*] and each FSA *i*, the observed rates, *M*_[*t*]*i*_ and *B*_[*t*]*i*_ are functions of (a) the effects, *α*, of FSA’s time-invariant socio-economic characteristics, (b) unobserved true heart health and over-indebtedness components, *m*_[*t*]*i*_ and *b*_[*t*]*i*_, and (c) measurement errors, *e*_*m*[*t*]*i*_ and *e*_*b*[*t*]*i*_ constrained to have equal variance *σ*_*em*_^2^ and *σ*_*eb*_^2^ across all years, except 2002. The equations with intercepts *μ*_*M*[*t*]_ and *μ*_*B*[*t*]_, to explain the measured rates are

$$ {M}_{\left[t\right]i}={\mu}_{M\left[t\right]}+{\alpha}_{me}{E}_i+{\alpha}_{mw}{W}_i+{\alpha}_{ma}{A}_i+{m}_{\left[t\right]i}+{e}_{m\left[t\right]i}, $$

$$ {B}_{\left[t\right]i}={\mu}_{B\left[t\right]}+{\alpha}_{be}{E}_i+{\alpha}_{bw}{W}_i+{\alpha}_{ba}{A}_i+{b}_{\left[t\right]i}+{e}_{b\left[t\right]i}. $$

Furthermore, the model specifies interrelations between the unobserved heart health and over-indebtedness components. Each latent variable at time [*t*] is a function of three components: (a) an autoregression, *β*, which represents the effect of the same factor at a previous time and is constant across lags; (b) a cross-lagged regression, *γ*, or the effect of the other variable at the previous time, also constant across lags, and (c) a disturbance, allowed to correlate with the disturbance for the other factor at the same year and constrained to have equal variance, *σ*_*dm*_^2^ and *σ*_*db*_^2^, and correlation, *ρ*_*mb*_^2^, across years. The explanatory equations for the factors are:

$$ {m}_{\left[t\right]i}={\beta}_m{m}_{\left[t-1\right]i}+{\gamma}_m{b}_{\left[t-1\right]i}+{d}_{m\left[t\right]i}, $$

$$ {b}_{\left[t\right]i}={\gamma}_b{m}_{\left[t-1\right]i}+{\beta}_b{b}_{\left[t-1\right]i}+{d}_{b\left[t\right]i}. $$

Additional assumptions are that the socio-economic characteristics, latent heart health and over-indebtedness components, measurement errors and disturbances were mutually uncorrelated. The variances of measurement errors *e*_*m*[2002]*i*_ and *e*_*b*[2002]*i*_ in year 2002 were allowed to differ from the error variances in subsequent years and latent variables for year 2002 were assumed to be correlated, to adjust for the unavailability of data before 2002.

The time frame of the study incorporates two economically-distinct periods: (1) an economically-stable period from 2002 to 2007 and (2) the economic recession of 2008–09. SEMs were therefore first fitted using 2002–07 data only, and then using entire data from 2002 to 09. When 2002–09 data were used, the SEM coefficients of the last two years were allowed to be different than the coefficients of 2002–07. This SEM feature was added to explore how the longitudinal association between AMI and BKC was affected by an adverse global economic event.

## Results and discussion

### Aggregate data

**Fig. 2:**
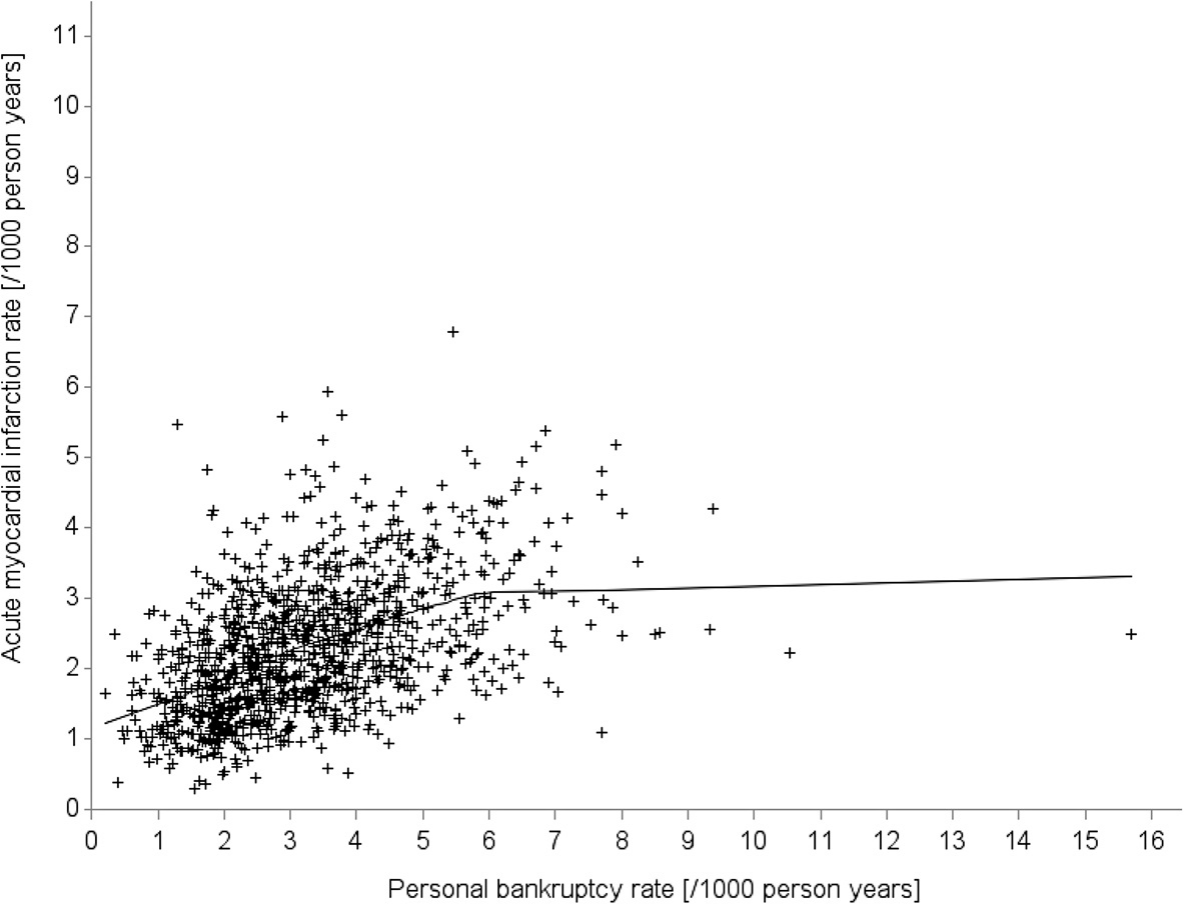
AMI and BKC rates. 8-year AMI rates by 8-year BKC rates for 1,155 Canadian forward sortation areas

Table 2 shows descriptive statistics, Pearson correlation coefficients between 8-year AMI rate, 8-year BKC rate and socio-economic characteristics. Across the 1,155 Canadian FSAs, included in the study, AMI and BKC rates were, on average, 2.27 and 3.23, respectively, per 1,000 person-year. Pearson correlation of 0.47 between AMI and BKC rates indicated a significantly positive relationship between these variables. Figure 2 displays a scatter plot of AMI and BKC rates for the years 2002–2009 for the 1,155 Canadian FSAs. The smoothed line can be interpreted as the unadjusted correlation of AMI and BKC rates and suggests a positive association between the two rates with a gradient that tends to decrease as BKC rate increases.

**Table 2:** 8-year AMI and BKC rates per 1,000 person-year and socio-economic characteristics. Pearson correlation coefficients and descriptive statistics for 1,155 Canadian forward sortation areas

Variables/Controlling factors	1	2	3	4	5	6	7	8	9	10
1. AMI rate	1									
2. BKC rate	.47**	1								
3. Median household income	−.52**	−.51**	1							
4. Employment rate^a^	−.63**	−.34**	.63**	1						
5. Unemployment rate^b^	.39**	.32**	−.47**	−.77**	1					
6. 65 years and over^c^	.67**	.15**	−.48**	−.64**	.17**	1				
7. University^d^	−.56**	−.40**	.38**	.31**	−.30**	−.20**	1			
8. No high school^e^	.52**	.32**	−.53**	−.54**	.58**	.22**	−.79**	1		
9. Home ownership^f^	.04	−.31**	.52**	.15**	−.08*	−.08*	−.26**	.03	1	
10. Single parent^g^	.29**	.54**	−.70**	−.42**	.32**	.27**	−.14**	.26**	−.70**	1
Mean	2.27	3.23	$59,591	62.4 %	7.1 %	18.0 %	22.1 %	24.0 %	74.0 %	25.6 %
STD	.94	1.55	$18,945	8.7 %	4.6 %	5.9 %	12.7 %	9.3 %	17.0 %	8.8 %

*P < .01, **P < .001

^a^Ratio of individuals 15 years and older who are employed to the population 15 years and older

^b^Ratio of individuals 15 years and older who are unemployed to the population 15 years and older

^c^Ratio of individuals 65 years and older to the population 20 years and older

^d^Ratio of individuals 15 years and older with university certificate, diploma or degree to the population 15 years and older

^e^Ratio of individuals 15 years and older with no high school diploma to the population 15 years and older

^f^Ratio of owned dwellings to the number of occupied private dwellings

^g^Ratio of single-parent families to the total number of families with children

All socio-economic characteristics, with the exception of home ownership, were significantly (*p* < 0.01), associated with both AMI and BKC rates, making them potential confounders of the observed AMI × BKC association. In addition any two socio-economic characteristics, with the exception of home ownership × no high school diploma were statistically significantly correlated, at a significance level lower than 0.01.

### Panel data

The parameters of several SEMs were estimated from the covariance matrix among the 19 measured variables (3 census variables, 8 yearly AMI rates and 8 yearly BKC rates). Table 3 summarizes the variables 19 × (19–1) / 2 = 171 correlations, means and standard deviations across 1,155 FSAs.

**Table 3:** Yearly AMI and BKC rates per 1000 person-year and socio-economic characteristics. Pearson correlation coefficients and descriptive statistics for 1155 Canadian forward sortation areas

Variables	1	2	3	4	5	6	7	8	9	10	11	12	13	14	15	16
1. 2002 AMI rate	1															
2. 2003 AMI rate	.68	1														
3. 2004 AMI rate	.66	.65	1													
4. 2005 AMI rate	.64	.65	.65	1												
5. 2006 AMI rate	.59	.63	.64	.66	1											
6. 2007 AMI rate	.55	.64	.60	.66	.67	1										
7. 2008 AMI rate	.54	.54	.62	.61	.60	.65	1									
8. 2009 AMI rate	.51	.55	.61	.58	.57	.65	.70	1								
9. 2002 BKC rate	.29	.33	.27	.30	.25	.25	.24	.20	1							
10. 2003 BKC rate	.29	.36	.25	.31	.27	.32	.26	.25	.76	1						
11. 2004 BKC rate	.34	.38	.33	.35	.32	.33	.32	.31	.72	.76	1					
12. 2005 BKC rate	.35	.37	.33	.35	.33	.37	.32	.31	.73	.75	.78	1				
13. 2006 BKC rate	.38	.41	.37	.37	.37	.38	.35	.35	.65	.65	.72	.79	1			
14. 2007 BKC rate	.33	.38	.40	.32	.36	.32	.34	.32	.57	.53	.61	.71	.74	1		
15. 2008 BKC rate	.38	.42	.36	.35	.32	.37	.33	.33	.55	.53	.64	.66	.71	.73	1	
16. 2009 BKC rate	.30	.35	.33	.33	.35	.33	.34	.30	.58	.56	.62	.67	.69	.73	.75	1
17. Education	−.30	−.33	−.35	−.37	−.40	−.42	−.44	−.46	−.28	−.31	−.34	−.39	−.38	−.35	−.34	−.39
18. Material wealth	−.12	−.15	−.09	−.09	−.06	−.04	−.03	.01	−.49	−.47	−.47	−.45	−.42	−.35	−.31	−.25
19. Working-age population	−.50	−.48	−.53	−.50	−.48	−.47	−.45	−.44	−.00	−.04	−.09	−.13	−.17	−.17	−.13	−.03
Mean	2.34	2.32	2.25	2.17	2.12	2.27	2.37	2.32	2.70	3.04	3.09	3.19	2.95	3.01	3.42	4.48
STD	1.18	1.14	1.16	1.15	1.13	1.15	1.16	1.17	1.59	1.70	1.69	1.82	1.71	1.85	1.97	2.32

Table 4 summarizes the SEM estimates based on 2002–07 panel data, only. In this table the four-columns correspond to four different SEMs fitted on data: (a) a reference model including both cross-lags, (b) a model with only a cross-lag from AMI to BKC, (c) a model with only a cross-lag from BKC to AMI and (d) a model with no cross-lags.

**Table 4:** Structural equation models for yearly AMI and BKC rates between 2002 and 2007. Parameter estimates and p-values

Parameters and fit indexes	AMI < = > BKC		AMI = > BKC		BKC = > AMI		No γ	
	AMI	BKC	AMI	BKC	AMI	BKC	AMI	BKC
Regression coefficients								
Autoregression (β)	.89 (<.01)	.89 (<.01)	.91 (<.01)	.89 (<.01)	.89 (<.01)	.91 (<.01)	.91 (<.01)	.91 (<.01)
Cross-lag (γ)	.015 (.02)	.07 (<.01)	-	.07 (<.01)	.014 (.03)	-	-	-
Education (α_e_)	−.43 (<.01)	−.56 (<.01)	−.43 (<.01)	−.57 (<.01)	−.43 (<.01)	−.56 (<.01)	−.43 (<.01)	−.57 (<.01)
Material wealth (α_w_)	−.09 (<.01)	−.74 (<.01)	−.1 (<.01)	−.74 (<.01)	−.09 (<.01)	−.75 (<.01)	−.09 (<.01)	−.74 (<.01)
Working-age population (α_a_)	−.57 (<.01)	−.14 (<.01)	−.57 (<.01)	−.13 (<.01)	−.58 (<.01)	−.14 (<.01)	−.58 (<.01)	−.13 (<.01)
Variances								
2002 factors (σ^2^_1_)	.47	1.36	.46	1.36	.47	1.34	.46	1.36
Disturbance 2003–2007 (σ^2^_d_)	.06	.36	.05	.36	.06	.35	.05	.35
Measurement error 2002 (σ^2^_e1_)	.43	.37	.44	.38	.42	.39	.44	.39
Measurement errors 2003–2007 (σ^2^_e_)	.41	.53	.41	.53	.41	.55	.41	.55
Covariances								
2002 factors (ρ_mb1_)	.29 (<.01)		.30 (<.01)		.30 (<.01)		.32 (<.01)	
Disturbances 2003–2007 (ρ_mb_)	−.008 (.31)		−.001 (.87)		.002 (.81)		.008 (.24)	
Goodness of fit								
Parameters	26		25		25		24	
Degrees of freedom	94		95		95		96	
Chi-square	479.3		484.7		489.6		494.6	
RMSEA	.0596		.0596		.06		.06	
CFI	.968		.967		.967		.967	
AIC	531.3		534.7		539.6		542.6	
Fit changes								
ΔΧ^2^/Δdf	na		5.4/1		10.3/1		15.3/2	
			na		4.9/0		9.9/1	
			na		na		5/1	

**Fig. 3:**
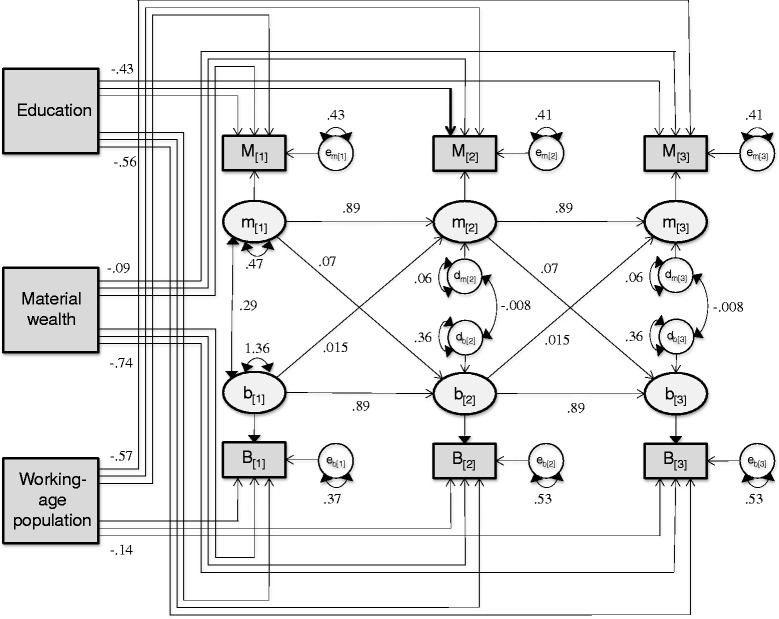
Diagram and estimates of SEM for AMI and BKC rates. Path diagram and parameter estimates of cross-lagged structural equation model for yearly AMI and BKC rates. Parameter estimates are based on 2002–07 data

Assessment of goodness-of-fit indices indicates that the model with both cross-lags is the most reasonable representation of these data. This model had both comparative fit index (CFI) and root mean square error of approximation (RMSEA) within acceptable limits (CFI > 0.93 [15], and RMSEA < 0.08 [16]). Figure 3 shows the path diagram for the first 3 waves of this SEM together with its parameter estimates computed based on 2002–07 data. Inspection of the parameters reveals high autocorrelation for both variables (0.89 for AMI and 0.89 for BKC), small cross-lagged regression coefficients from BKC to AMI (0.015) and from AMI to BKC (0.07), high initial variance for both variables (0.47 for AMI and 1.36 for BKC), small (0.06) and moderate (0.36) disturbances in AMI and BKC, respectively, that do not appear to be correlated (ρ_mb_ = −0.008). In addition, education, material wealth and working-age population were shown to be negatively associated with the measured rates.

In summary the longitudinal association between AMI and BKC was estimated as follows: (1) an increase of 100 in BKC count is associated with an increase of 1.5 (*p* = 0.02) in the AMI event count in the following year and (2) an increase of 100 in the AMI event count is associated with an increase of 7 (*p* < 0.01) in the BKC count, in the following year.

Table 5 summarizes the estimates based on entire 2002–09 data. Two cross-lagged models are shown: (a) the reference model with parameters (autoregressive, cross-lags, disturbance variances and correlations) constrained to be equal across all 8 years from 2002 to 2009 and (b) the reference model with parameters freed to take distinct values in years 2008 and 2009. Compared to reference SEM fit on 2002–07 data, the constrained 2002–09 model had similar parameter estimates, however, poorer fit as indicated by RMSEA and CFI. The 2002–09 model with freed parameters for the last two years revealed that the association BKC × Following-year AMI was not present (γ = .015 and .002, p > .50) and AMI × Following-year BKC was increased (γ = .24 and .30, *p* < .01) during the recession of 2008–09. The fit of this model was comparable to the fit of 2002–07 SEM.

**Table 5:** Structural equation models for yearly AMI and BKC rates between 2002 and 2009. Parameter estimates and p-values

Parameters and fit indexes	AMI < = > BKC		AMI < = > BKC	
	AMI	BKC	AMI	BKC
Regression coefficients				
Autoregression 2002–2007 (β)	.88 (<.01)	.96 (<.01)	.88 (<.01)	.91 (<.01)
Autoregression 2008 (β)	─״─	─״─	.87 (<.01)	1.05 (<.01)
Autoregression 2009 (β)	─״─	─״─	.96 (<.01)	1.08 (<.01)
Cross-lag 2002–2007 (γ)	.013 (<.01)	.08 (<.01)	.015 (.01)	.05 (.01)
Cross-lag 2008 (γ)	─״─	─״─	.015 (.50)	.24 (<.01)
Cross-lag 2009 (γ)	─״─	─״─	.002 (.89)	.30 (<.01)
Education (α_e_)	−.43 (<.01)	−.55 (<.01)	−.43 (<.01)	−.55 (<.01)
Material wealth (α_w_)	−.09 (<.01)	−.75 (<.01)	−.09 (<.01)	−.75 (<.01)
Working-age population (α_a_)	−.54 (<.01)	−.06 (.07)	−.57 (<.01)	−.15 (<.01)
Variances				
2002 factors (σ^2^_1_)	.48	1.16	.48	1.31
Disturbance 2003–2007 (σ^2^_d_)	.08	.32	.06	.29
Disturbance 2008 (σ^2^_d_)	─״─	─״─	.15	.39
Disturbance 2009 (σ^2^_d_)	─״─	─״─	.03	.82
Measurement error 2002 (σ^2^_e1_)	.41	.46	.42	.41
Measurement errors 2003–2009 (σ^2^_e_)	.40	.68	.40	.62
Covariances				
2002 factors (ρ_mb1_)	.26 (<.01)		.28 (<.01)	
Disturbances 2003–2007 (ρ_mb_)	−.005 (.42)		−.005 (.46)	
Disturbances 2008 (ρ_mb_)	─״─		−.02 (.36)	
Disturbances 2009 (ρ_mb_)	─״─		−.07 (.03)	
Goodness of fit				
Parameters	26		40	
Degrees of freedom	164		150	
Chi-square	1061.3		800.7	
RMSEA	.073		.061	
CFI	.945		.96	
AIC	1113.3		880.7	
Fit changes				
ΔΧ^2^/Δdf	na		260.6/14	

## Conclusions

Our panel data analysis at the FSA level indicates that an increase in the number of personal bankruptcy filings is associated with an increase in the number of AMI events, in the following year and an increase in the number of AMI events is associated with an increase in the number of personal bankruptcy filings, in the following year. These temporal associations are explained only partially by socio-economic factors.

The fact that BKC was associated with AMI in the following-year supports the hypothesis that financial and debt stress negatively impacts heart health. This is consistent with findings from the health poll conducted at the height of the recession in 2008 by the Associated Press [17] in the US, showing 6 % of those with high debt stress reported heart attacks, double the rate of those with low debt stress. Moreover since this association was not solely explained by confounding socio-economic factors, it may indicate that a region’s count of bankruptcy filings adds extra information to the region’s median household income and other conventional measures of socio-economic deprivation and permits a yet another explanation of the heart disease occurrence within a region. An important advantage of bankruptcy filing counts is the yearly availability of data, as opposed to socio-economic characteristics that are assessed every five years, when census data are collected. These facts make yearly regional bankruptcy filing rate a valuable predictor to be added to established socio-deprivation indices for better understanding of a region’s heart disease occurrence and planning of regional health resources. During the recession years 2008 and 2009, personal bankruptcy was not associated with heart health. This is in accordance with previous results [18, 19] obtained from US data, that the economic activity had almost no effect on health during the last recession.

The reverse, AMI being associated with following-year BKC was also identified in our analysis; supporting the hypothesis that heart attack interferes with employability of affected individuals [20–23] and could provoke debt problems. For the financial crisis years, 2008 and 2009, this association was estimated to values higher than those in previous years 2002–2007, indicating that an adverse health event, like AMI, could aggravate debt problems more in a recession than in an economically-stable environment.

### Limitations

A common practice in medical literature is to standardize the AMI rate for age, since age is known to be a major risk factor for AMI. We choose not to standardize the AMI counts and rates for age, because the scope of our analysis was to assess the association of AMI with BKC, a variable that could not be standardized for age due to the lack of age records in the BKC data. However, we attempted age-adjustment by including the census age indicators, like percentage of elderly aged 65 and over or employment and unemployment rates in the SEM. As discussed, this method is similar to including in a regression model the confounder correlated with both dependent and independent variable [24].

Only the 2006 population counts were available for 1,155 FSAs included in the study. The 2006 counts were used as proxies for the population counts in the remaining years of our study period. The yearly AMI and BKC rates may have been affected by bias by using the incorrect denominator. However, we expect the bias in the AMI and BKC rates for 2002–09 to be small, because 2006 was the midpoint of our study period.

The median yearly population size of a Canadian FSA is large, amounting to 13,000 individuals aged 20 years and over and causing high inter-subject variability within FSA. Hence, our yearly rates exhibited over-dispersion across FSAs and indicated that they may not follow the normal distribution. In spite of this aspect of the data, we choose SEMs, models that assume data normality to estimate parameters, because of SEM’s flexibility to model panel data and ability to estimate longitudinal associations.

Many factors could affect AMI and BKC rates. We strived to account for the effects of these factors by incorporating the census variables in our model. However our established positive temporal associations AMI × Following-year BKC and BKC × Following-year AMI may be the result of an ecological fallacy.

We selected BKC rate, or rate of legally bankrupt borrowers, because it is a variable easy to measure at the aggregate level and is available on a calendar year basis. Though, an objective measure of debt-burden of an area, BKC represents a limited understanding of over-indebtedness, mostly selecting severe cases of over-indebtedness and missing the perceived burden of debt [25].
